# Precision Reprogramming in CAR-T Cell Therapy: Innovations, Challenges, and Future Directions of Advanced Gene Editing

**DOI:** 10.7150/ijbs.124144

**Published:** 2025-10-24

**Authors:** Zimo Jia, Jiajin Wu, Jiyue Zhang, Peixian Zheng, Haoxuan Zhang, Yiqin Lin, Tao Pan, Meng Wu, Yuqin Song

**Affiliations:** Key Laboratory of Carcinogenesis and Translational Research (Ministry of Education), Department of Lymphoma, Peking University Cancer Hospital & Institute. Beijing 100142, China.

**Keywords:** CAR-T, CRISPR/Cas9, gene editing, immunotherapy

## Abstract

Chimeric antigen receptor (CAR)-T cell therapy represents a breakthrough in cancer immunotherapy, demonstrating impressive clinical outcomes, particularly for hematologic malignancies. However, its broader therapeutic application, especially against solid tumors, remains limited. Key challenges include T cell exhaustion, limited persistence, cytokine-mediated toxicities, and logistical hurdles associated with manufacturing autologous products. Emerging gene editing technologies, such as CRISPR/Cas systems, base editing, and prime editing, offer novel approaches to optimize CAR-T cells, aiming to enhance efficacy while managing toxicity and improving accessibility. This review comprehensively examines the current landscape of these gene editing tools in CAR-T cell therapy, highlighting the latest advancements, persisting challenges, and future directions. Leveraging gene editing holds the potential to transform CAR-T therapy into a more potent, safer, and broadly applicable modality for cancer and beyond.

## 1. Introduction

Minimizing recurrence and durable remission have always been the central objectives of oncology research. Immunotherapy has gradually made its mark in the field of cancer treatment, which lies in rejuvenating the exhausted and synergetic state of tumor-killing immune cells and thwarting the immune-evasion tactics of cancer cells, with the goal of eliciting a robust and effective anti-tumor response 1. Recent innovations in immunotherapeutic strategies, ranging from immune checkpoint inhibitors (ICIs) to adoptive cellular therapy (ACT) and cancer vaccines, have reshaped cancer treatment paradigms and produced remarkable clinical progress.

Among these advances, chimeric antigen receptor (CAR) technology represents a major milestone. CARs are engineered receptors designed to redirect T cells toward tumor-associated antigens (TAAs) in a major histocompatibility complex (MHC)-independent fashion, facilitating tumor eradication. CAR-T cells (CAR-Ts) targeting the pan-B-cell marker CD19 have exhibited unprecedented response rates in refractory B-cell malignancies 2. While several CAR-T therapies have received FDA approval for hematological cancers, their application to solid tumors remains primarily investigational, with significant clinical adoption hindered by challenges such as suboptimal efficacy, safety concerns, restricted patient accessibility, and prohibitive manufacturing costs 3, 4.

The remarkable clinical achievements of CAR-T cell therapy (CAR-Tct) are inextricably traced back to advancements in genetic engineering, specifically in engraftment, persistence, and proliferation of CAR-Ts derived from large-scale *in vitro* cultures—key factors for sustained therapeutic responses 5, 6. Among the available genetic editing tools, the CRISPR/Cas9 system has emerged as pivotal for developing next-generation or allogeneic CAR-Ts with enhanced biosafety and efficacy profiles. In this review, we scrutinize novel CRISPR/Cas9-based cancer immunotherapy scenarios, situating them within the context of cutting-edge advances in immunotherapy, while also addressing the persistent challenges that have hindered substantial clinical success. Furthermore, we delineate prevailing trends and propose viable strategies to overcome these barriers. Ultimately, this paper aims to catalyze transformative advances in immunotherapy.

## 2. Current challenges

**Figure 1:**
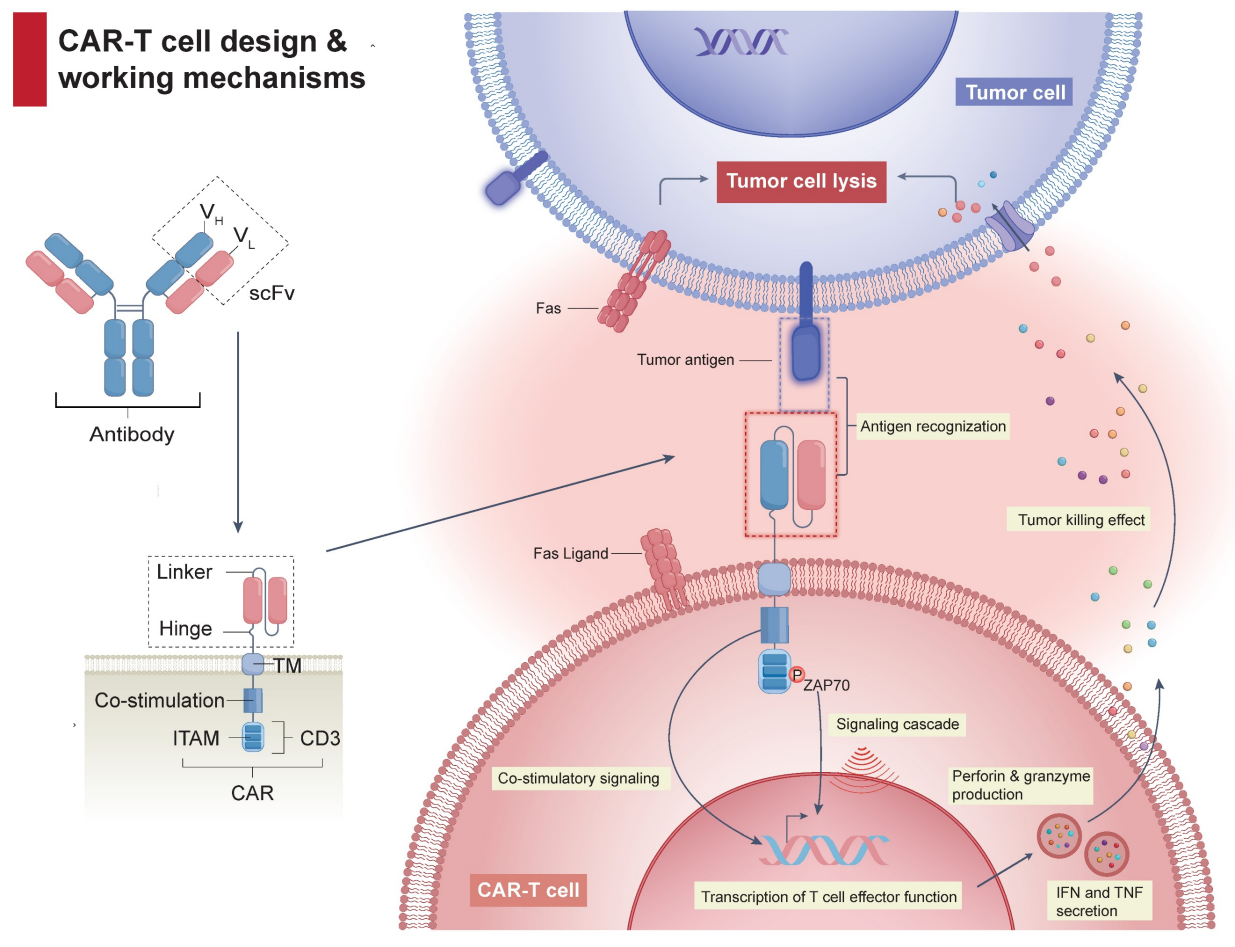
** CAR-T cell architecture and anti-tumor mechanisms.** A typical CAR-T cell expresses a chimeric receptor composed of an extracellular scFv, linked via a hinge and transmembrane region to intracellular co-stimulatory and CD3ζ signaling domains. Upon binding to its target antigen, CAR engagement leads to clustering and phosphorylation of ITAMs within the CD3ζ domain, which recruits ZAP-70 and initiates downstream signaling cascades. This ultimately results in T cell activation, cytokine secretion, and targeted tumor cell killing.

**Figure 2:**
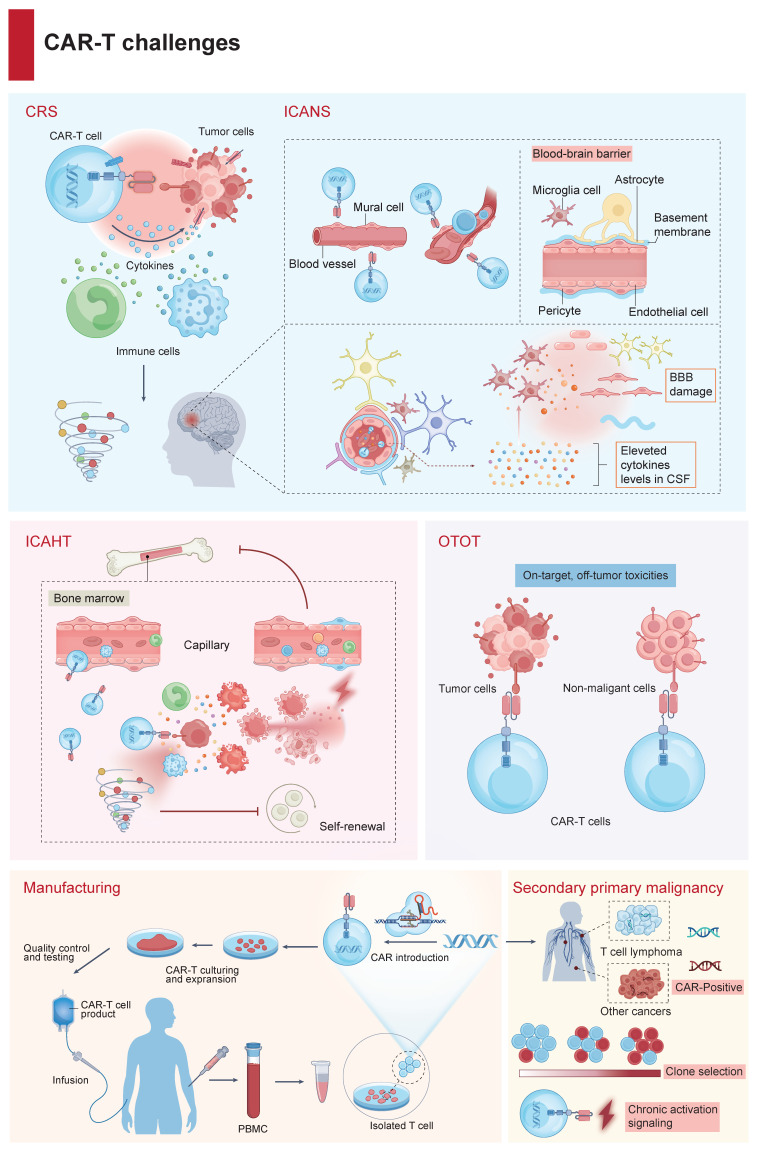
** Key challenges in CAR-T cell therapy.** CAR-Tct faces several key challenges: CRS resulting from excessive immune activation; ICANS, primarily caused by BBB dysfunction and central nervous system inflammation; ICAHT, stemming from on-target recognition of antigens expressed on hematopoietic progenitors and the inflammation in bone marrow microenvironment; OTOT, which occurs when healthy tissues expressing the target antigen are inadvertently targeted; a complex, multi-step manufacturing process spanning from leukapheresis to CAR transduction, expansion, and reinfusion; and the risk of second primary malignancies following CAR-T treatment.

Despite revolutionizing the management of hematological malignancies, CAR-Tct faces several critical obstacles limiting broader clinical implementation. The complexity of CAR design (Figure 1), incorporating antigen-binding (AD), hinge (HD), transmembrane (TMD), and signaling domains (SD), underpins both its efficacy and its risks. Achieving an optimal balance between effectiveness and manageable toxicity, along with addressing logistical hurdles, remains challenging (Figure 2).

### 2.1 CRS

Cytokine release syndrome (CRS) is a common and sometimes life-threatening complication, occurring in 42-93% and 84-95% of patients receiving CD19 7 or BCMA CAR-T therapies 8, respectively. It results from excessive cytokine and chemokine release, with IL-6 being a central mediator 9. IL-6 blockade agents, such as tocilizumab and siltuximab, effectively control CRS symptoms but may inadequately prevent, or even exacerbate, neurotoxicity (immune effector cell-associated neurotoxicity syndrome, ICANS) 10, 11. Emerging strategies, such as preemptive administration of IL-6-binding agents (IL-6 'sponges') and higher-frequency dosing of IL-1 inhibitors like anakinra, represent potential avenues for CRS mitigation. Moreover, single-cell RNA sequencing (scRNA-seq) analyses have identified IFN-γ-regulated inflammatory signatures and IL-1-associated resistance pathways, suggesting new therapeutic targets, although preclinical studies indicate potential impacts on CAR-T expansion and function 12, 13.

### 2.2 ICANS

ICANS is another significant toxicity, presenting as a spectrum of neurologic symptoms and often occurring alongside or subsequently to CRS (Figure 2) 14. The pathogenesis may involve loosening of the blood-brain barrier (BBB) owing to endothelial disruption and a systemic inflammatory state enables the intracerebral passage of both circulating cytokines and CAR-Ts, followed by glial cell injury 15. Mild ICANS is primarily managed with supportive care and close neurological monitoring. For moderate to severe ICANS, corticosteroids—such as dexamethasone or methylprednisolone—are the first-line therapy according to ASCO and ASTCT guidelines 16, 17. Tocilizumab, while effective for CRS, is not recommended for isolated ICANS. High-throughput proteomic analyses have identified IL-18 as being associated with the onset of ICANS symptoms, suggesting that targeting the IL-18 pathway may represent a potential strategy to reduce neurotoxicity 10, 18. However, the efficacy of IL-18 antagonists in preventing or treating ICANS remains to be confirmed in preclinical or clinical studies. Concurrently, novel CAR designs are being developed to minimize the risks of CRS and ICANS while enhancing tumor antigen recognition and effective T-cell signaling.

### 2.3 ICAHT

Emerging clinical insights have cast a spotlight on cytopenia, a frequently encountered and insidious complication of CAR-Tct, now categorized as immune effector cell-associated hematotoxicity (ICAHT, Figure 2) 19. ICAHT is intricately linked to the severity and protraction of neutropenia, with late ICAHT denoting neutropenia that lingers beyond one-month post-infusion 20. The CAR-HEMATOTOX model, which integrates variables reflective of hematopoietic reserves—encompassing baseline hemoglobin, platelet, and neutrophil counts, alongside baseline serum ferritin and CRP, has demonstrated effective in predicting delayed ICAHT and infection risk 21. As the management landscape for ICAHT continues to evolve, models like CAR-HEMATOTOX hold the potential to guide preemptive strategies, including the judicious deployment of G-CSF and tailored anti-infective regimens, allowing for tailored treatments during early ICAHT 22. In scenarios of prolonged cytopenia, where autologous stem cells have been cryopreserved, autologous stem cell augmentation has shown feasibility following both CD19- and BCMA-targeted CAR-Tct. For few patients (<5%) in whom late ICAHT remains recalcitrant, allogeneic hematopoietic stem cell transplantation (HSCT) constitutes the ultima ratio 23.

### 2.4 OTOT

CAR-Ts targeting antigens shared with normal tissues can induce severe, sometimes fatal, toxicity in healthy organs (OTOT), particularly in solid tumor settings (Figure 2). A case in point is the application of CD19 CAR-Ts, which, while adept at eradicating malignant B cells in ALL, inadvertently ensnare normal B cells in their therapeutic crosshairs 24. A subset of mural cells, indispensable for the maintenance of BBB integrity, become unintended casualties owing to their expression of CD19, resulting in BBB disruption and contributing to observed toxicities 25. The scarcity of truly tumor-specific surface antigens (neoantigens) makes target selection challenging. Nevertheless, most targets in solid tumors are TAAs, such as EGFR, CAIX, and HER2, which are also expressed on healthy tissues 26. Neoantigens, particularly those expressed on the cell surface, are a rarity, especially in tumors characterized by a low mutational burden 27. Instances of severe toxicities have been reported in patients receiving CAR-Tct targeting TAAs: fatal pulmonary toxicity with CAR-Ts against HER2, lung toxicity with CEA-targeting, hepatic toxicity with CAIX-directed, and dermal toxicity with EGFR- targeting 28, 29.

### 2.5 Manufacturing and accessibility

Most approved CAR-T products are autologous, whose bespoke manufacturing limits scalability and can delay access (Figure 2). Against this backdrop, allogeneic, “off-the-shelf” universal CAR-T (UCAR-T) products manufactured from healthy donors and stored as ready-to-infuse doses offer several practical advantages: they can shorten the vein-to-vein time, lower cost via economies of scale, and improve lot-to-lot consistency. UCAR-T is also a critical alternative for patients in whom autologous manufacturing is not feasible—such as those with T-cell malignancies or with poor T-cell fitness after heavy pretreatment—and the on-hand inventory can facilitate redosing when initial expansion is suboptimal, potentially improving overall efficacy. Currently, this strategy is still maturing and requires extensive gene editing and mitigation of risks associated with graft rejection, immunogenicity, and graft-versus-host disease (GVHD) 30. Early clinical trials have yielded encouraging signals (e.g., anti-CD19/CD7 programs for leukemia and BCMA-directed products for myeloma), but autologous CAR-T remains the most practical and clinically validated option at present 31-33. Furthermore, high-dimensional profiling of CAR-T manufacturing underscores that culture conditions strongly shape cell phenotype and function, often more than the integration site itself. Early (day 5) products retain stem-like, metabolically active T cells with high proliferative capacity, whereas prolonged culture (day 10) enriches for terminally differentiated, potentially exhausted subsets; while both show similar cytotoxicity, they differ in activation and checkpoint profiles. Cryopreservation modestly alters some memory/metabolic markers but preserves overall function 34. These findings underscore the importance of optimizing manufacturing protocols to maintain favorable metabolic and phenotypic traits, supporting improved CAR-T cell efficacy and accessibility.

### 2.6 CAR-T‑associated malignancies

Although rare, several reports have documented second primary malignancies (SPMs) following CAR-Tct, implying the potential toxicity of CAR-T products (Figure 2). Notably, among 22 cases reported by the FDA, three exhibited integration of the CAR transgene within malignant clones, one of which involved insertion into the 3'-UTR of PBX2, an oncogene implicated in lymphomagenesis following treatment with anti-BCMA autologous CAR-Tct 35, 36. However, subsequent sequencing revealed that oncogenic mutations pre‑existed CAR-T manufacturing, making causality ambiguous. Mechanistically, T‑cell transformation may result either from insertional mutagenesis disrupting tumor suppressors or activating proto‑oncogenes, or from prolonged CAR and endogenous TCR signaling prompting accumulation of mutational events. Alternatively, the rare occurrences might reflect expansion of pre‑malignant T‑cell clones inadvertently harvested and modified during CAR-T production 36. While FDA safety reviews underscore the “remarkably low” risk with autologous CAR-Ts, the finding of SPMs in CAR-T treated patients remains concerning 37. Taken together, these clinical cases and molecular findings highlight the theoretical risk of oncogenic transformation—whether via insertional mutagenesis, clonal selection, or chronic activation signaling—underscoring the importance of long‑term genomic surveillance in CAR-T cell recipients.

## 3. Gene editing

To surmount these hurdles, advanced gene-editing techniques have emerged as indispensable tools. Foremost among these is CRISPR/Cas9, renowned for its precision and versatility in engineering cellular genomes to enhance therapeutic efficacy and safety. Subsequent sections delve deeper into innovative CRISPR-based methodologies and highlight sophisticated approaches such as base editing and prime editing, illuminating their promising roles in refining CAR-T cell therapeutics (Table 1).

### 3.1 CRISPR/Cas9

**Figure 3:**
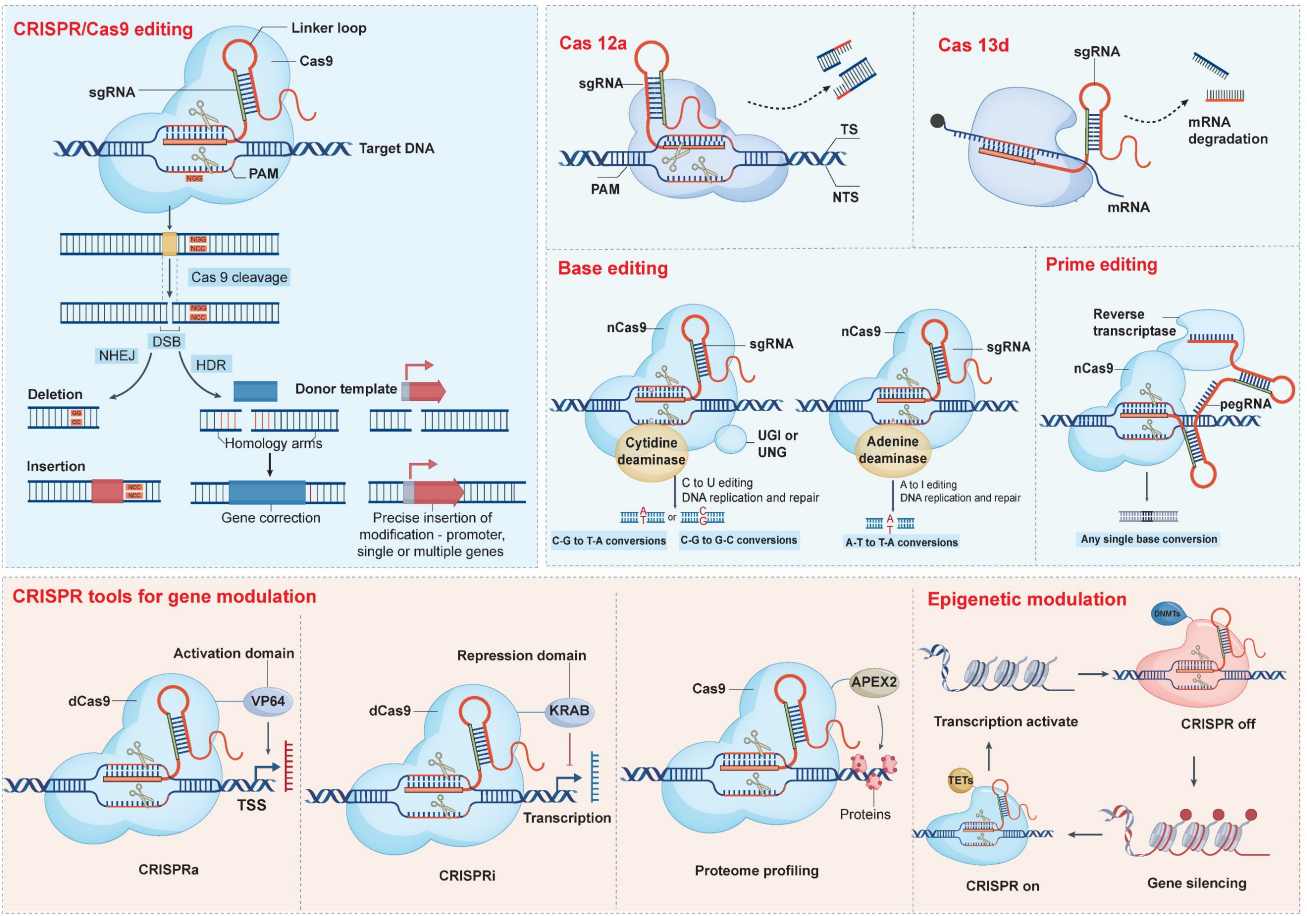
** CRISPR-based editing and modulation platforms.** CRISPR technology encompasses a versatile toolbox: Cas9 nuclease creates DNA DSBs for indels (via NHEJ) or precise insertions (via HDR), while Cas12a introduces staggered cuts and Cas13d targets RNA for degradation. Base editors—cytidine or adenine deaminases fused to inactive Cas9—enable direct C→T or A→G conversions, and prime editors (nCas9-reverse transcriptase with pegRNA) install virtually any single-base change without DSBs. Beyond editing, dCas9 fused to VP64 or KRAB domains drives gene activation (CRISPRa) or repression (CRISPRi), Cas9-APEX2 facilitates proteome profiling, and epigenetic effectors (TETs or DNMTs) tethered to Cas9 allow locus-specific DNA demethylation or silencing.

CRISPR/Cas9 system enables precise cleavage of specific genomic loci in human cells and supports a wide range of transgene insertions, including expression markers, selectable reporters, gene expression regulators, and even the integration of entirely new gene cassettes 38, 39 (Figure 3). Nuclease-deficient Cas9 (dCas9), which lacks endonuclease activity and can be conjugated with transcriptional repressors or activators to create CRISPR interference (CRISPRi) and CRISPR activation (CRISPRa) systems, respectively. Targeted epigenetic modifications are also available within the CRISPR/Cas9 family, although the journey toward ideal orchestration is fraught with challenges. CRISPR off induces stable epigenetic silencing, which in some contexts can be reversed by CRISPR on, providing a dynamic and valuable addition to the existing CRISPR toolkit 40.

### 3.2 Base and prime editing

In contrast to conventional CRISPR/Cas9-mediated gene editing, base editors (BEs) afford targeted nucleotide substitutions without instigating DSBs or necessitating donor DNA, thus circumventing error-prone repair pathways. Typically, BEs incorporate a Cas9 nickase (nCas9) tethered to a deaminase enzyme, occasionally complemented by ancillary domains designed to heighten editing precision (Table 1) 41. The pioneering BEs, cytosine base editors (CBEs) and adenine base editors (ABEs), orchestrate CG-to-TA and AT-to-GC nucleotide conversions, respectively 42, 43. As numerous genetic disorders stem from discrete nucleotide mutations, BEs represent a compelling therapeutic strategy for rectifying such aberrations. The clinical validation of this approach is underscored by FDA approval of therapies such as Casgevy and Lyfgenia for sickle cell disease. Nevertheless, the adoption of base editing techniques confronts certain hurdles, notably the risk of unintended bystander edits within a restricted nucleotide editing window, necessitating continual refinements to bolster specificity and curtail off-target effects 44, 45.

With greater flexibility, prime editors (PEs) can meticulously target and amend virtually any genomic sequence, free of DSBs reliance (Table 1). Beyond simple base alterations, PEs excel in the insertion or removal of short DNA stretches at designated sites. Prime editing has been successfully demonstrated in various organisms, including plants, mice, and organoid lines, achieving extremely low levels of off-target edits 46, 47.

## 4. Application of gene editing in CAR-Tct

**Figure 4:**
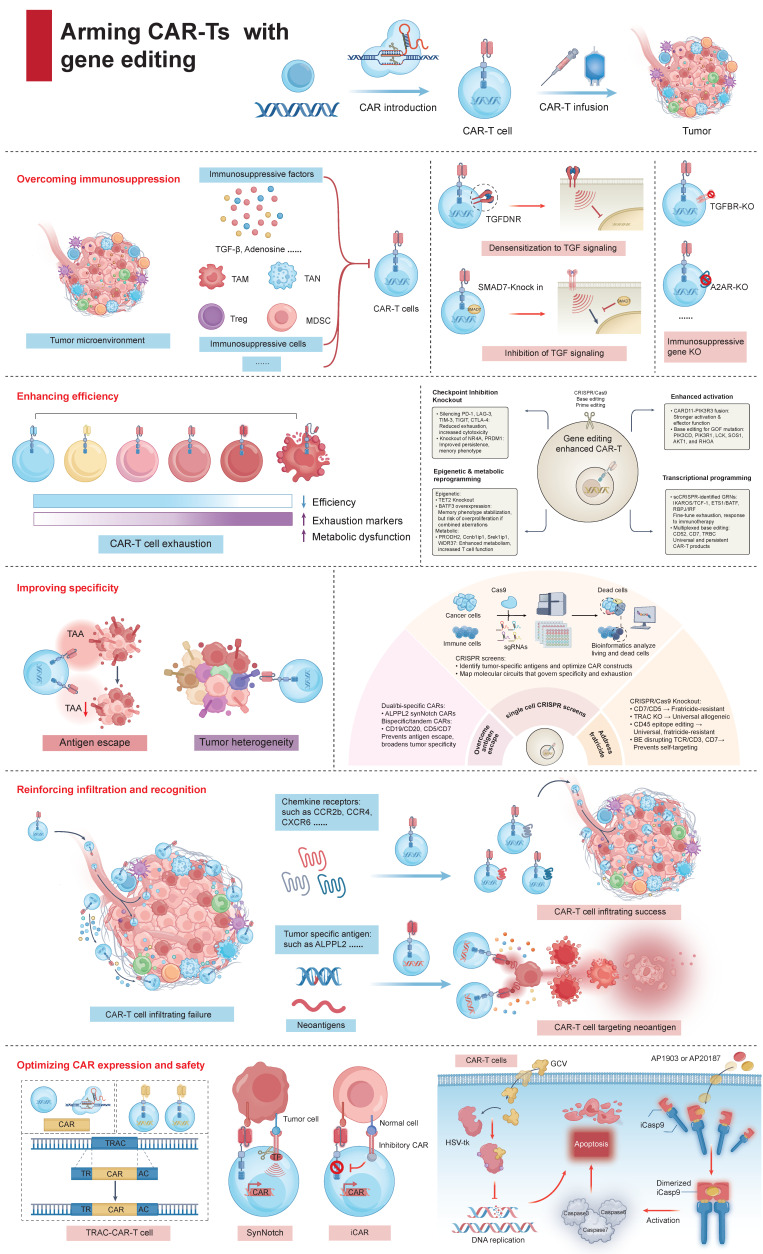
** Arming CAR-Ts with gene editing for improved cancer immunotherapy.** Gene editing enhances CAR-T cell therapy through multiple strategies: (1) Overcoming immunosuppression by disrupting inhibitory pathways and targeting suppressive cells in the tumor microenvironment. (2) Enhancing efficiency by knocking out exhaustion-related genes and reprogramming epigenetic and metabolic pathways to improve persistence and cytotoxicity. (3) Improving specificity with fratricide-resistant CAR-T cells (CD7 or CD5 knockout), bispecific/tandem CARs, and epitope editing to prevent antigen escape and off-target effects. (4) Reinforcing infiltration and recognition by engineering chemokine receptors (e.g., CCR2b, CXCR6) and targeting neoantigens, thus promoting CAR-T cell trafficking and tumor targeting. (5) Optimizing CAR expression and safety via precise CAR insertion, synthetic Notch/inhibitory CARs, and safety switches (including HSV-tk and iCas9). Together, these approaches enable the next generation of CAR-T therapies with greater efficacy, selectivity, and safety.

In oncological settings, patients often exhibit compromised immune systems, which include phenotypic alterations and functional deficits in T cells, significantly undermining anti-tumor immunity. The emergence of genome editing technologies, such as CRISPR/Cas9 system, base editing tools, and prime editing agents, have opened new frontiers in reshaping and potentiating human T cells. In this section, we have discussed the major translational barriers of CAR-Tct and how gene editing strategies address these issues (Figure 4).

### 4.1 Overcoming immunosuppression

#### 4.1.1 CRISPR/Cas9 remove the 'immunosuppressive chains'

The tumor microenvironment (TME) resembles a paradoxical arena for fueling and dousing tumor immunity. Regrettably, the rise of an immunosuppressive TME (ITME) profoundly undermines the effectiveness of immunotherapies 48. Deciphering and manipulating the immunosuppressive networks within the ITME to cultivate a conducive TME represents an advancement in scaling up the success of contemporary CAR-Tct. One promising tactic entail combating inhibitory cytokines prevalent in the ITME, with TGF-β being a principal adversary. Presently, multifaceted approaches have formulated to neutralize TGF-β's inhibitory effects 49. Tang et al. introduced dominant-negative TGF-β receptors that bind TGF-β without transmitting its inhibitory signal, rendering the cells impervious to TGF-β-mediated suppression 50. Alternatively, CAR-Ts can be modified to produce TGF-β-neutralizing antibodies or trap proteins, effectively reducing local concentrations of TGF-β. Moreover, CRISPR/Cas9 technology allows the disruption of TGFBR2, or the introduction of suppressors like SMAD7, thereafter enhancing CAR-T resistance to ITME-induced exhaustion 51, 52. Surprisingly, TGF-β within the ITME can be repurposed from a hindrance into a stimulant. This is exemplified by the use of chimeric switch receptors, which fuse the extracellular domain of the TGF-β receptor to the intracellular signaling domain of a co-stimulatory molecule 53. Upon engaging TGF-β, these modified receptors convert an immunosuppressive cue into an immunostimulatory one, thereby enhancing CAR-T cell function even amidst high TGF-β levels (Figure 4). And bispecific CAR-Ts aim to simultaneously address TGF-β-mediated suppression and recognizing tumor antigens. CAR-Ts can also be engineered to secrete cytokines such as IL-7 and CCL19, which counteract the deleterious effects of TGF-β 54.

Otherwise, IL‑12 has re‑emerged via control circuits that confine its expression to the tumor (e.g., hypoxia‑responsive or activation‑inducible knock‑ins), as well as mesothelin/MUC16 programs exploring regional delivery in early‑phase trials; together these data support context‑restricted IL‑12 as a potent, clinically tractable amplifier of antigen‑directed killing. In parallel, gene editing of adenosine signaling (e.g., ADORA2A/A2A receptor knockout) renders CAR-T cells refractory to adenosine-driven suppression arising from the extracellular ATP-to-adenosine cascade, and edits to CD39/CD73 or enzymatic adenosine catabolism are under active evaluation 55, 56.

Within in the ITME, malignant cells hijack various infiltrating immune cells, encompassing regulatory T cells (Tregs), myeloid-derived suppressor cells (MDSCs), tumor-associated macrophages (TAMs) and neutrophils (TANs), to subvert anti-tumor immunity. Substantial efforts have been dedicated to reprogramming these suppressive cell populations to fuel tumor-antagonizing responses. CAFs-derived SDF-1α lures CXCR4+ MDSCs into the tumor milieu, which subsequently elicit apoptosis in CD8+ T cells (CD8Ts) and curtail the lytic function of CAR-Ts 57. Targeting CAFs can, in principle, deliver a dual benefit—blunting CAF-driven invasion and resistance while also diminishing MDSC infiltration. Yet eradication of CAFs in pancreatic cancer models has paradoxically accelerated disease progression 58, highlighting the functional heterogeneity of CAF subsets and the hazards of indiscriminate depletion. Accordingly, therapeutic efforts should prioritize phenotype modulation—reprogramming or restraining pathogenic CAF states—rather than wholesale clearance. In line with this microenvironment-centric strategy, CAR-Ts engineered to co-express CXCR4 demonstrate superior antitumor activity, with improved stromal trafficking and concomitant reductions in MDSC ingress, yielding more durable tumor control than conventional CAR T cells 59. Restriction of STAT3 signaling in CXCR4 CAR-Ts decreases the levels of TNF-α, IL-17A, and IL-6, obstructs SDF-1α expression in an NF-κB-dependent fashion, and consequently impedes the MDSC recruitment into tumor site. The immunosuppressive influences exerted by TAMs presents another barrier to effective PC therapy 60. CAR-Ts targeting CD123 or F4/80 can eradicate TAMs and retard tumor progression 61, 62. Recent studies indicate that CAR-Ts directed against TAMs and TAM receptors—specifically TYRO3, AXL, and MERTK—can significantly decelerate tumor growth 63, 64. Similarly, removing other immunosuppressive cells presents an enticing therapeutic avenue.

#### 4.1.2 CRISPR/Cas9-based functional genomics to address immunosuppression

CRISPR/Cas9 screens in primary T cells have uncovered inhibitory regulators (such as MED12 and CCNC) that, once ablated, markedly amplify antigen-specific proliferation and cytotoxic potency across diverse CAR-T constructs 65. Further expansive CRISPR screening in human CD8Ts has spotlighted additional inhibitory molecules, including CBLB, RASA2, SOCS1, and TCEB2, whose disruption therapeutically fortifies CAR-T proliferation and cytotoxicity 66. CBLB have been reported to regulate T cell activation thresholds and energy 67, whose effect on exhaustion is suggested but not confirmed. CBLB deficiency propels CD8^+^ T cell-mediated tumor clearance 68. Simultaneously, Carnevale et al. illuminated RASA2's role as a brake on antigen responsiveness, showing that its deletion amplified CAR-T's cytotoxic prowess and tenacity across preclinical malignancy models 69. Equally compelling, SOCS1 emerges as a non-redundant intrinsic inhibitor curbing T-cell activity and functional breadth *in vivo*, whose removal robustly rejuvenates both CD4^+^ and CD8^+^ T-cell responses 70, 71. Disruption of SOCS1's SH2 domain substantially boosts IL-12 sensitivity, IL-2 responsiveness, and overall anti-tumor efficacy in CAR-Ts 71. Translational research on human CD19-targeted CAR-Ts confirms improved functionality and vitality upon SOCS1 suppression 72.

Beyond immune cell-intrinsic factors, CRISPR screening in tumor cells has identified critical vulnerabilities that can be exploited to counteract immunosuppression. For instance, PTPN2, a phosphatase that dampens IFN-γ signaling through dephosphorylation of STAT1 and JAK1, negatively regulates tumor antigen presentation and impedes cytokine-driven tumor inhibition 73-75. Eliminating PTPN2 potentiates IFN-γ signaling, augments antigen presentation, and intensifies cytokine-driven tumor growth inhibition, suggesting potential therapeutic benefits from its inhibition 73. Similarly, receptor-interacting protein kinases (RIPKs) such as RIPK1 and RIPK2 orchestrate immune evasion and targeting RIPK1 reduces recruitment of immunosuppressive ARG1+ myeloid cells and primes tumors for immune attack 76-79, while RIPK2 deficiency disrupts the desmoplastic TME, ushers in an upsurge in MHC-I surface presentation by curtailing the NBR1-mediated pathway of autophagy-lysosomal degradation, and sensitizes tumors to ICB, resulting extended survival 80. RIPK3, frequently silenced by oncogenes such as including AXL, BRAF, and notably, MYC, which limits necroptosis by impairing RIPK1-RIPK3 interplays 81.

#### 4.1.3 CRISPR/Cas9 rewires cytokine networks to counteract immunosuppressive barriers

Modulation of IFN-γ pathway further shapes the TME and CAR-T function. For instance, murine Qa-1b (encoded by H2-T23), homolog to human HLA-E, is upregulated by IFN-γ receptor signaling and contributes to tumor resistance before and during CAR-Tct 82. Blocking Qa-1b's inhibitory receptor, NKG2A, enhances therapeutic efficacy, corroborated by *in vivo* studies in PDAC mouse models where Qa-1b suppression sensitized tumors to ICB therapy 83. IFN-γ production by NK cells during CAR-T therapy can paradoxically drive both beneficial and inhibitory effects—enhancing endogenous immunity while inducing Qa-1b-mediated resistance 84. Compounding this complexity, tumor-intrinsic IFN-γ-receptor signaling shapes CAR-T susceptibility in a context-dependent manner. Across available datasets, loss-of-function alterations in IFNGR1 or its downstream kinases JAK1/JAK2 exert little measurable impact on leukemia/lymphoma responses to CAR-Ts, yet in solid tumors—including glioblastoma—abrogation of this axis consistently promotes resistance. Mechanistically, intact IFN-γ signaling amplifies a suite of tumor programs that cooperate with CAR-T activity—enhanced antigen processing/presentation, upregulation of adhesion and death-receptor pathways (e.g., ICAM-1, FAS), and chemokine remodeling—whereas pathway loss diminishes immunologic visibility and T-cell engagement, blunting cytotoxicity 85. This divergence argues for routine profiling of the IFN-γ pathway and for combination strategies that restore or bypass IFN-γ responsiveness in solid-tumor settings.

#### 4.1.4 Leveraging base editing to disarm immunosuppressive pathways in CAR-Ts

BE technologies are emerging as powerful tools to enhance CAR-T cell resistance to immunosuppressive cues in the TME. Moreover, ABE targeting the N74 glycosylated residue of PD1 effectively downregulates PD-1 expression in CAR-Ts, resulting in enhanced cytotoxic activity both *in vitro and in vivo* 86. Pule et al. developed a novel protocol to generate circular BE RNA (circBE) instead of traditional linear BE mRNA, resulting in clinical dose CAR-Ts with lower PD1 expression pattern 87. Collectively, these advancements highlight the transformative potential of BEs for the precise, safe, and efficient engineering of CAR-Ts for cancer treatment.

### 4.2 Enhancing efficiency

Long-term persistence of CAR-T cell function is crucial for durable anti-tumor immunity. Gene editing approaches have focused on reprogramming T cell metabolism and transcriptional states to favor the generation and maintenance of memory T cell (T_MEM_) within the nutrient-deprived, suppressive TME (Figure 4).

#### 4.2.1 CRISPR/Cas9 approaches to overcome exhaustion and boost memory formation in CAR-Ts

The potency of CAR-Ts can be augmented by silencing genes that make them vulnerable to inhibitory signals. For example, CAR-Ts lacking NR4A demonstrated superior tumor regression in mice 88. Pulling from cancer's playbook, Roybal et al. integrated 71 mutations—identified in neoplastic T cells—into CAR-Ts, with the fusion CARD11-PIK3R3 standing out 89. This amalgamation intensifies signaling through the CBM complex, a linchpin for T cell activation and functionality during antigen recognition 90. In multiple cancer-bearing mouse models, tumor burdens diminished markedly. Despite the specter of these powered-up CAR-Ts metamorphosing into malignancies, animal studies have yet to stoke the fires of safety apprehensions. Additionally, the potency improvement permits lower dosage and obviated the necessity for lymphodepleting chemotherapy, which carries risks of mutagenesis and secondary malignancies 91.

Typically, the curtailed lifespan of CAR-Ts is ascribed to the onset of exhaustion states, which constitutes a significant impediment to their therapeutic efficacy 92, 93. In patients achieving favorable remissions, the infused CAR-Ts generally manifest lower levels of exhaustion markers (e.g., PD-1, LAG-3, and TIM-3) 94. These features predominantly represent ICs that compel CTLs to lapse into states of dormancy or exhaustion. Several critical drivers have been ascertained. For instance, the engagement of PD-L1 with PD-1 controls multiple potential destinies for activated CD8Ts, encompassing anergy, exhaustion, and apoptosis 95. Yet PD-1 is also a state marker of recent antigen encounter: in defined settings, PD-1^high^ CAR-Ts display superior immediate cytotoxicity and antitumor protection relative to PD-1^low^ counterparts, while adoptive transfer of PD-1^high^ cells alone fails to achieve durable tumor control, highlighting a distinction between short-term effector capacity and long-term fitness 96. Tumor context further modulates checkpoint biology; in ovarian cancer, intracellular (rather than surface) PD-L1 is enriched within cytotoxic T cells, offering a plausible explanation for the muted activity of conventional PD-1/PD-L1-directed agents in this setting 97. Veritably, the concurrent administration of CAR-Tct and anti-PD-1 antibodies has yielded encouraging outcomes in patients 98. Furthermore, enhanced anti-tumor efficacy has also been observed in preclinical studies through the silencing of the PD-1 axis within CAR-Ts via CRISPR/Cas9 99. Given the pivotal roles of other inhibitory immune checkpoint receptors, such as LAG-3, TIM-3, and TIGIT, in concert with PD-1 function, concurrent intervention of multiple pathways is projected to further enhance the CAR-T performance. Comparative studies show that dual PD-1/TIGIT suppression yields a distinctive synergy that surpasses PD-1 silencing alone, whereas pairing PD-1 with TIM-3, LAG-3, or CTLA-4 fails to provide incremental benefit 100. Mechanistically, PD-1 deletion primarily amplifies acute effector function, while TIGIT inhibition constrains terminal differentiation and transcriptional exhaustion, preserving a stem-like pool that sustains responses—together accounting for the observed synergy 100. This concept has advanced to the clinic, with a phase 1/2 trial of PD-1/TIGIT-edited CD19 CAR-T in adults with relapsed/refractory DLBCL (NCT04836507). By contrast, CTLA-4 editing illustrates the context dependence of multiplex strategies: loss of CTLA-4 unleashes CD28 costimulation and stabilizes CAR surface expression under high antigen load, improving tumor control when targeted alone, yet this benefit is not recapitulated when CTLA-4 and PD-1 are co-edited—pointing to non-additive or even countervailing circuit interactions 101. Collectively, these data argue for mechanism-guided, indication-specific checkpoint engineering rather than indiscriminate stacking, with PD-1/TIGIT emerging as a leading axis and CTLA-4 manipulation reserved for settings dominated by CD28-driven activation.

#### 4.2.2 Targeting epigenetic and metabolic pathways in CAR-Ts via CRISPR/Cas9

Reprogramming epigenetic signatures to stabilize memory phenotypes offers additional avenues for extending CAR-T persistence. For example, knockout of TET2, involved in DNA demethylation, promotes robust clonal expansion with a durable memory profile 102. TET2 knockout induces a central memory phenotype, fostering clonal expansion of CAR-Ts. Despite the observed long-term remission in a patient with chronic lymphocytic leukemia using TET2-deficient CAR-Ts, research has shown that biallelic TET2 disruption, coupled with sustained expression of factors like BATF3, can precipitate excessive proliferation in an antigen-independent fashion in CAR-Ts 103, 104. Enforced BATF3 expression programs CAR-Ts toward a memory-like state and counteracts transcriptional and epigenetic features of exhaustion 105. Importantly, BATF3 overexpression alone does not elicit adverse effects in T cells, yet risk becomes context-dependent: sustained BATF3 in combination with high-risk genetic backgrounds (for example, biallelic TET2 loss) can drive antigen-independent clonal expansion, and in malignant T-cell contexts BATF3 cooperates with IRF4 or engages an IL-2R super-enhancer module, conferring oncogenic properties 106, 107. These argue for activation-linked or titratable BATF3 designs that preserve its memory-promoting and anti-exhaustion benefits while avoiding constitutive, high-level expression in permissive genomic contexts.

Metabolic reprogramming has been highlighted by genome-wide CRISPR screens in CD8Ts, which identified genes such as PRODH2, Ccnb1ip1, Srek1ip1, and WDR37 as positive regulators of T cell degranulation and function 108, 109. Chief among them, PRODH2 emerged as a pivotal player in amplifying cancer cell lethality. Elevated PRODH2 reprogrammed T cell metabolism, invigorating T cell vitality and tumoricidal capacity 109. The story underpinning PRODH2's influence on T cell functionality appears to lie in its recalibration of proline metabolism, a crucial player in T cell anti-tumor influence 110. Although the impact of PRODH2 on T_MEM_ differentiation remains largely unexplored, modulation of metabolic pathways holds great promise for extending CAR-T persistence. Further investigations have explored the roles of nutrient signaling pathways in memory formation. For instance, amino acid transporters Slc7a1 and Slc38a2 were found to hinder T_MEM_ differentiation by activating mTORC1 signaling, while targeting these transporters or the GDP-fucose-Pofut1-Notch axis can selectively enhance T_MEM_ development 111.

#### 4.2.3 Leveraging scCRISPR screens to reprogram CAR-T cell fate and longevity

Transcriptional programming is another avenue for enhancing CAR-T efficiency. Single-cell CRISPR (scCRISPR) has enabled the dissection of gene regulatory networks (GRNs) underlying T cell fate. Zhou et al. have leveraged scCRISPR screens to reconstruct the GRNs governing the fate of CD8+ cytotoxic T lymphocytes (CTLs) in cancer 112. They revealed three key transcriptional axes (IKAROS/TCF-1, ETS1/BATF, and RBPJ/IRF) that shape CTL heterogeneity, each unfurling new therapeutic vistas 112. IKAROS, encoded by IKZF1, plays a nuanced role: while it facilitates the maturation of precursor exhausted T cells (Tpex) into fully exhausted T (Tex) through TCF-1 modulation, it also restrains metabolic and mTORC1 activity to prevent excessive differentiation 113. IKZF1 loss impairs transition from Tpex to Tex, possibly by affecting metabolic competence 113. In contrast, by manipulating BATF, ETS1 curtails mTORC1 activity and metabolic rewiring, thus steering the differentiation of Tpex towards Tex. ETS1 disruption bolsters anti-tumor immunity and ICB efficacy 113. Elevated RBPJ levels links to terminal CTL exhaustion and hyperresponsiveness to immunotherapies, marking RBPJ as a potential target for reprogramming Tex cells and in synergy with ICB. The underlying mechanism involves NOTCH-independent RBPJ signature that hampers IRF1 function 114, 115. These findings offer insights into reprogramming CTL fates, promising advancements in CAR-T efficacy.

In clinical settings, CRISPR screening elucidates resistance pathways and refines therapeutic strategies. For instance, PRRX2 was identified as central to androgen receptor inhibitor resistance in prostate cancer, amenable to reversal via BCL2 and CDK4/6 inhibitors (CDK4/6i) 116. Moreover, TGFβ3 serves as a predictive biomarker in TNBC for palbociclib therapy, where combination with CDK4/6i treatment demonstrates enhanced anti-proliferative synergy, suggesting innovative approaches for overcoming therapeutic resistance 117, 118.

#### 4.2.4 Base editing-driven enhancement of CAR-T fitness and longevity

The application of high-throughput base editing is rapidly advancing CAR-T engineering, especially in generating universal and highly persistent CAR-T products. Multiplexed base editing, for instance, has facilitated the concurrent disruption of CD52, CD7, and TRBC loci, paving the way for the creation of universally deployable CD7-targeting CAR-Ts (BE-CAR7) 119. Clinical application of BE-CAR7 has led to molecular remission and successful immune reconstitution in patients with refractory T-cell ALL, underscoring the clinical potential of BE platforms.

Further, BE technology allows for the introduction of gain-of-function mutations into genes central to T cell activation and persistence. High-throughput BE screening have facilitated the generation of thousands of clinically significant variants across critical genes, including PIK3CD, PIK3R1, LCK, SOS1, AKT1, and RHOA 120. Specifically, BE-induced gain-of-function (GOF) mutations in PIK3CD and PIK3R1 in T cells, including those engineered with a melanoma-specific T cell receptor or in various generations of CD19 CAR-Ts, lead to enhanced signaling, cytokine production, and the ability to effectively kill melanoma and leukemia cells 120. These GOF mutations, unlike loss-of-function (LOF) mutations or silent controls, contributed to improved CAR-T cell efficacy in leukemia cell killing and cytokine production, demonstrating the potential of BE to optimize CAR-Tct and improve their clinical outcomes.

### 4.3 Improving specificity

The specificity of CAR-Ts is central to minimizing off-target effects and maximizing anti-tumor selectivity. Gene editing technologies have enabled the refinement of targeting strategies and the prevention of undesirable T cell-T cell interactions (fratricide), as well as the mapping of molecular circuits that govern specificity and exhaustion (Figure 4).

#### 4.3.1 Leveraging CRISPR/Cas9 for antigen-specific CAR-T engineering and fratricide resistance

A significant challenge in developing CAR-T therapies for T cell malignancies is the phenomenon of fratricide—the mutual destruction of CAR-Ts that occurs when their target antigen is shared between malignant and healthy T cells, including the CAR-T product itself. To overcome this, CRISPR/Cas9 have been leveraged to knock out endogenous T cell antigens, allowing for the production of CAR-Ts resistant to fratricide. CD7 and CD5 are two well-characterized targets in this context. CD7 is broadly expressed on T-cell acute lymphoblastic leukemia (T-ALL) cells as well as on normal T cells, leading to self-recognition and rapid elimination of CAR-Ts unless the antigen is ablated. Recent studies have demonstrated that CRISPR-mediated knockout of CD7 (CD7KO) in donor T cells enables efficient generation of CD7-targeted CAR-T products that are resistant to fratricide, expand robustly *in vitro*, and exhibit potent anti-leukemic activity in preclinical models 121. Early-phase clinical trials have shown that CD7KO CAR-Ts are not only feasible to manufacture at scale but can also mediate significant anti-tumor responses in patients with relapsed/refractory T-ALL, while avoiding severe T cell aplasia 122, 123. Similarly, CD5 is another pan-T cell marker frequently targeted in T cell leukemias and lymphomas. Knockout of CD5 (CD5KO) using CRISPR/Cas9 or TALENs prior to CAR transduction preserves CAR-T cell viability during manufacturing and prevents fratricidal killing upon antigen engagement. Studies have shown that CD5KO CAR-Ts retain cytotoxicity against malignant CD5+ T cells while maintaining an early memory phenotype, increased expansion capacity, and favorable *in vivo* persistence 124, 125. Notably, Ottaviano et al. demonstrated in a first-in-human phase I trial that CD5KO CD5-CAR-Ts can be manufactured efficiently, are safe, and induce durable remissions in patients with T cell lymphoblastic leukemia 125.

Beyond CD5 and CD7, knockout of other lineage markers (e.g., TRAC for universal allogeneic CAR-Ts) 126 and the use of safety switches are also under investigation to further improve the manufacturing and clinical performance of CAR-T therapies for T cell malignancies. Antigen knockout approaches such as CD5KO and CD7KO represent a pivotal innovation in the field of T cell malignancy immunotherapy, allowing for the scalable generation of potent, persistent, and fratricide-resistant CAR-T cell products.

Additionally, CRISPR-based forward genetic screens in tumor cells have accelerated the identification of candidate neoantigens and immunomodulatory targets, supporting the development of more selective and potent CAR constructs. High-throughput screening not only uncovers antigens with tumor specificity but also identifies molecules that enhance T cell function and resistance to exhaustion. scCRISPR screening has provided granular insight into the regulatory axes that influence specificity and exhaustion.

#### 4.3.2 CRISPR/Cas9 enables dual-target CAR-T engineering to overcome antigen escape

The limited availability of true tumor-specific antigens (TSAs) and the frequent antigen-loss escape seen with tumor-associated antigens (e.g., mesothelin) constrain single-target CAR-T efficacy (Figure 4). To counter antigen loss and heterogeneity, dual-target strategies have gained traction. These are implemented as tandem/bispecific CARs, bicistronic “dual-CAR” designs, or synNotch/logic circuits 127. By requiring recognition of two antigens, these formats lower the probability that tumor clones can evade killing through down-modulation of a single target, broaden the recognition spectrum across heterogeneous tumors, and—when logic gating is used—can raise activation thresholds to improve functional selectivity and reduce on-target/off-tumor engagement.

Clinical experience in hematologic malignancies supports these concepts: programs such as CD19/CD22 and CD19/CD37 have reported high response rates with fewer antigen-negative relapses relative to single-antigen approaches 128, 129. For example, tandem CD19/CD20 CAR-T (tanCAR-T) mitigates target downregulation, and a single-arm phase I/II study showed meaningful activity in patients relapsing after CD19 CAR-T 130; similarly, dual CD20/CD19 products have yielded encouraging outcomes with >80% objective responses in some cohorts 131. In solid tumors, where TSAs are scarce, pairing a context sensor with a killing CAR can improve selectivity: ALPPL2 has emerged as a TSA in ovarian cancer and mesothelioma 132, and embedding ALPPL2 sensing within synNotch circuits limits tonic signaling and favors a memory-like state while subsequently driving CARs against HER2, mesothelin, or MCAM, improving control in preclinical models 133.

Dual-targeting is not a panacea. Simultaneous or convergent down-regulation of both targets—although less likely—can still mediate escape; AND/NOT-gate designs may become inactive if one required antigen is lost. Therapeutic efficacy remains dependent on *in vivo* persistence, which is curtailed by exhaustion and the ITME. Also, engineering and producing dual-target products increases vector size/complexity, places higher demands on potency and quality-control assays (viability, dual-arm expression, and function). Furthermore, extending these strategies to T-cell malignancies introduces unique challenges, notably fratricidal effects due to shared antigen expression among CAR-Ts 121. In an innovative study, researchers employed CRISPR/Cas9 technology to excise CD5 and CD7 during the production of bispecific CARs, resulting in fratricide-resistant fully human CD5/CD7 bispecific CAR-Ts that exhibit powerful anti-tumor activity against T-cell malignancies 134. Interestingly, tandem CD5/CD7 CARs maintain cytolytic durability, showcasing superior lysis of CD7-tumor cells than dual CAR constructs 134. Although complete T-cell aplasia has not been uniformly reported, the risk of profound immunodeficiency warrants vigilance. In selected high-risk settings, bridging allogeneic HSCT after achieving deep molecular remission with CD5/CD7 bispecific CAR-T may be considered; prospective studies are needed to define its role.

#### 4.3.3 Refining CAR-T cell specificity through base and prime editing of antigenic epitopes

BE has been harnessed to engineer precise modifications in epitopes of the pan-leukocyte antigen CD45, a crucial target in UCAR-T 135. The introduction of function-preserving mutations via BE has yielded epitope- edited CD45 CAR-Ts resistant to fratricide yet capable of exerting robust anti-tumor effects against diverse hematologic malignancies 135. Furthermore, when applied to HSCs, this epitope editing confers protection from CAR-T-mediated OTOT while preserving the physiological functions of CD45, thereby establishing a safe and versatile foundation for CD45-directed immunotherapeutic interventions 135.

Fratricide, which results from CAR-Ts recognizing antigens expressed on their own surface, remains a challenge in T cell malignancy therapy. To address the problem of CAR-T 'fratricide', where shared antigens such as CD3 and CD7 lead to mutual CAR-T cell attacking, BEs have been employed to disrupt TCR/CD3 and CD7 by introducing stop codons or eliminating splice sites 136. This results in fratricide-resistant CAR-Ts that retain potent anti-leukemia activity with no detectable off-target effects on CAR-T specificity 136. Furthermore, prime editing has demonstrated value in the precise modification of other clinically relevant antigens. Through meticulous optimization, the PE represents a dramatic improvement in editing efficiency and product purity. Zhang et al harnessed PEs to modify the CD123 epitope on HSCs and progenitor cells, preserving essential protein expression and cellular functions critical for hematopoietic integrity 137. This targeted editing confers robust protection to healthy cells against CAR-T-mediated cytotoxicity. The PE- engineered HSPCs were resistant to CAR-T cell lysis *in vivo and in vitro* for treating relapsed AML 137. Despite these remarkable advances, BE and PE technologies still face certain limitations compared to conventional CRISPR/Cas9 editing. These include lower editing efficiency in some cell types and constraints related to the range of targetable sequences. Ongoing advances are expected to further improve their applicability and safety profiles in CAR-T cell engineering.

### 4.4 Reinforcing infiltration

Insufficient infiltration of CAR-Ts into solid tumors continues to impede therapeutic efficacy. Chemokine gradients constructed by neoplastic cells often serve as beacons, guiding immune cells toward malignant foci 138. Equipping CAR-Ts to express tailored chemokine receptors (CCRs) responsive to these gradients holds promise for stimulating their tumor-homing capacity 139 (Figure 4).

### 4.4.1 CRISPR/Cas9 improves CAR-T homing and infiltration in solid tumors

Chemokine CCL2 is broadly expressed across various malignancies. CCR2b, the cognate receptor for CCL2, has demonstrated a remarkable propensity to navigate towards CCL2-enriched microenvironments, thereby mobilizing CAR-Ts permeating into high-CCL2-expressing malignancies such as neuroblastoma and melanoma 140. Functional analyses further underscore the enhanced migratory capacity conferred by CCR2b and CCR4 expression in mesothelin-targeted CAR-Ts 141. These CCR-engineered CAR-Ts demonstrate elevated cytotoxicity and robust secretion of cytokines such as IL-2, IFN-γ, and TNF-α 141. Additionally, concurrent expression of IL-7 and CCR2b significantly promotes trafficking and persistence of GD2-specific CAR-Ts 142. While CCR2b improves migration, there is a risk it could lead CAR-Ts to normal tissues as well, which can cause off-target effects and damage healthy cells. Brain metastases are traditionally deemed an insurmountable fortress for adoptively transferred CAR-Ts due to the BBB. Yet, the detection of CCL2 gradients in both primary and brain-metastatic NSCLC sheds light on exploiting this chemotactic signal to accelerate CAR-T infiltration across the BBB 143. Employing CRISPR/Cas9-mediated insertion of a CCR2b expression module into B7-H3-targeted CAR-Ts notably augmented their selective migration and tumor-specific activity, achieving sustained regression of cancerous lesions without harming adjacent healthy brain tissue 144. Despite these advances, caution is warranted. CAR-Ts engineered with CXCR2, receptive to a plethora of chemokines, have yielded only marginal improvements in anti-tumor activity. This limited efficacy is compounded by the propensity of these cells to traverse into a broad array of normal tissues 144. Thus, fine-tuning CCR expression in CAR-Ts requires careful consideration of the balance between therapeutic benefits and potential adverse outcomes.

#### 4.4.2 CRISPR/Cas9 rewires intrinsic signaling

Beyond adding homing receptors, CRISPR can improve infiltration by editing genes that gate IFN-γ responsiveness and chemokine networks on either the T-cell or tumor side. In tumor cells, CRISPR loss-of-function of PTPN2 amplifies IFN-γ-STAT1 signaling and upregulates CXCL9/10/11 and CCL5, which increases T-cell recruitment and sensitizes tumors to adoptive T-cell therapies; complementary CRISPR deletion of PTPN2 in engineered T cells enhances effector function and persistence, providing a bidirectional route to boost trafficking and function in solid tumors 145. While safety and durability require careful evaluation, these CRISPR perturbations outline tractable nodes to raise chemokine gradients and facilitate deeper CAR-T ingress into immune-excluded tumors.

### 4.5 Optimizing CAR expression and safety

Effective activation of T cells necessitates TCR engagement with antigen presentation and costimulatory signals. However, the expression of endogenous TCR interfere transgenic receptors in CAR-Ts, disrupting signaling cascades and cellular trafficking. TCR excision, a maneuver enhancing the specificity and activity of CAR-Ts, has shown promise in mice models 146 (Figure 4).

#### 4.5.1 Precision control of CAR expression via CRISPR/Cas9

CAR transgene via CRISPR/Cas9 at the TRAC locus in human T cells, effectively silencing endogenous TCR expression while placing CAR expression under the baton of the native promoter 147, 148. This elegant move addresses two longstanding issues of CAR-Tct: the capriciousness of CAR levels and the disruptive influence of endogenous TCR activity. The result is a notable improvement in performance, particularly in ALL mouses 148. Furthermore, this approach mitigates tonic CAR signaling, facilitates efficient CAR internalization and re-expression of CARs following antigen exposure, and delays premature effector T-cell differentiation and exhaustion. Innovative designs to fine-tune CAR-T activation thresholds is endless. Rogelio et al. developed a dual-recognition mechanism amalgamating low- and high-affinity ultrasensitive circuits, enabling cytotoxic T cells to discriminate targets antigen densities via a sigmoidal response curve 149. Initially, a low-affinity receptor serves as an antigen-sensitive gate, priming cells for subsequent transcriptional activation upon encountering high-density HER2 expression, thus sparking CAR expression and aggrandizing both proliferative and cytotoxic responses 149.

Aimed at conquering CAR-T toxicities, cell lysis and apoptosis can be ignited by calling up specific suicide genes with death switches. This affords the precision to deactivate therapeutic cells upon unforeseen expansion or prophylactically, in scenarios where biomarkers like IFN-γ, IL-13, and MIP1α indicate impending adverse events 150. Beyond, it may also shed light on who will suffer from relapse or adverse effects from CAR-Tct. Two primary suicide platforms are the herpes simplex virus thymidine kinase (HSV-tk) system and inducible caspase 9 (iCasp9) 151. The former operates by phosphorylating certain nucleoside analogues to generate GCV triphosphate that suppresses DNA synthesis and triggers cell death. The later exploits an inducible caspase 9 safety switch (e.g., AP1903, AP20187 as inducers) to selectively eradicate suicide gene-enriched cells (Figure 4) 152-154. Alternative strategies involving NK cell recognition of CD20 or EGFRt, although effective, draw additional genetic materials and arouse effector signals externally, which may breed immunogenic responses and suboptimal transgene expression 155.

To refine CAR expression control further, Patterson et al. handled Cas9 ribonucleoproteins to target exon 1 of the UMPS gene, a key index for auxotrophic cell growth 156. This nutrient metabolism-directed CAR design allows CAR-T cell activity to be meticulously regulated through uridine metabolism. In parallel, Kwong et al. have developed photothermal-sensitive intra-tumoral CAR-Ts, incorporating a synthetic gene switch that responds to mild temperature stimuli (40-42°C) 157. When these cells are activated by gold nanorods *in vivo*, they secrete super-agonist IL-15 and are further equipped with a bispecific T cell engager targeting the NKG2D receptor. This design enables precise redirection of CAR-Ts towards antigen-negative tumors under thermal control. Concurrently, a focused ultrasound (FUS) device, capable of being initiated by short-pulsed FUS in vitro, has been integrated into the CAR backbone with high specificity 158. When applied to primary tumor sites in murine models, MRI-guided FUS selectively heats the environment to 42°C, triggering CAR-T activation that targets malignant cells while sparing adjacent healthy tissues 158. This approach of direct FUS control over inducible CAR-T activity *in vivo* presents a groundbreaking, non-invasive therapeutic option for solid tumors. Complementing these sophisticated control mechanisms, reversible 'ON' and 'OFF' switches for CAR-Ts have been drafted, ruled by small molecules like resveratrol, lenalidomide, dasatinib, rimiducid, and rapamycin. For instance, CAR-Ts outfitted with a resveratrol-responsive transcriptional regulatory device can achieve controllable anti-cancer cytotoxicity, balancing therapeutic potency with safety through a resveratrol-titratable system 159.

#### 4.5.2 Base editing platforms for site-specific and safe CAR transgene integration

A critical aspect of safe and effective CAR-T therapy is the site-specific and stable expression of the CAR transgene. The RNA aptamer-driven Pin-point BE system offers a highly efficient and accurate approach for performing gene knockouts and integrating transgenes at specific sites in T cells 160. When compared to CRISPR/Cas9, this BE platform enables simultaneous multiplex gene knockouts and CAR integration in a single step while exhibiting superior editing efficiency, higher purity, and a substantially lower incidence of chromosomal translocations 160. This research highlights the potential of the Pin-point BE system to achieve more precise and effective genome editing in advanced cellular therapies. Furthermore, integrating BE with CRISPR/Cas9 nuclease-mediated knock-in techniques enables the targeted insertion of CAR constructs into the TRAC locus, alongside knockout of MHC class I and II genes 86. The resulting engineered CAR-Ts, which lack both TCR and MHC molecules, exhibit resistance to allogeneic T cell attacks *in vitro* 86. Together, these advances illustrate how base and prime editing are reshaping the landscape of CAR transgene engineering—maximizing safety, precision, and therapeutic flexibility.

## 5. Current challenges of Cas9-based CAR-T engineering

Integrating CRISPR/Cas9 into the vanguard of CAR-Tcts heralds a shift, permitting unparalleled precision in the bespoke tailoring of T cells by enabling insert and/or remove pertinent molecular elements (Figure 4). This confers upon CAR-Ts an augmented therapeutic response. Numerous studies have validated the safety and practicability of multiplex CRISPR/Cas9 engineering in T cells for oncological applications. Nowadays, a proliferation of base research and clinical trials exploring CRISPR/Cas9-fortified CAR-T modalities is unfolding. We have characterized the key current gene-edited CAR-T products at the clinical-stages (Table 2).

Concerns regarding genotoxicity and long-term safety remain significant obstacles to clinical translation. The most salient risks include off-target DNA editing, chromosomal rearrangements, and possible oncogenic transformation (Table 1). CRISPR/Cas9 relies on sgRNA-directed recognition of target DNA sequences. However, imperfect complementarity can result in DSBs at unintended genomic loci, leading to off-target indels or larger genomic alterations. These off-target mutations can disrupt tumor suppressor genes or activate proto-oncogenes, raising the risk of malignant transformation. Advances such as high-fidelity Cas9 variants and improved guide design algorithms have reduced off-target rates, yet comprehensive genome-wide assessment remains necessary for each product 161. Multiplex gene editing, increasingly used to engineer allogeneic or “stealth” CAR-Ts, involves simultaneous DSBs at multiple loci. This significantly increases the risk of chromosomal translocations, large deletions, or other structural variants 162. Recent clinical and preclinical studies have reported detectable frequencies of chromosomal translocations in triple- or quadruple-edited T cells 163, 164. While the functional impact of low-frequency rearrangements is unclear, rare cases of CAR-T-related subsequent T-cell malignancies have been described 35, 37, underscoring the need for robust screening strategies. There is emerging evidence linking genome-editing-related genotoxicity with the development of secondary malignancies after CAR-Tct 37. Mechanistically, these events may involve insertional mutagenesis, off-target effects in proto-oncogenes, or persistent chromosomal instability. Longitudinal monitoring and molecular tracking are now increasingly recommended as part of post-infusion surveillance in clinical trials.

To address these challenges, the field is adopting safer editing approaches (e.g., base editing, prime editing), transient delivery formats (RNP or mRNA), high-fidelity nucleases, and rigorous cell product release criteria, including genome-wide genotoxicity assessment and clonal tracking. Nonetheless, given the potentially irreversible nature of genomic changes, cautious interpretation of early clinical success is warranted, and longer follow-up is essential.

## 6. Novel technological platforms and innovations in CAR-Tct

**Figure 5:**
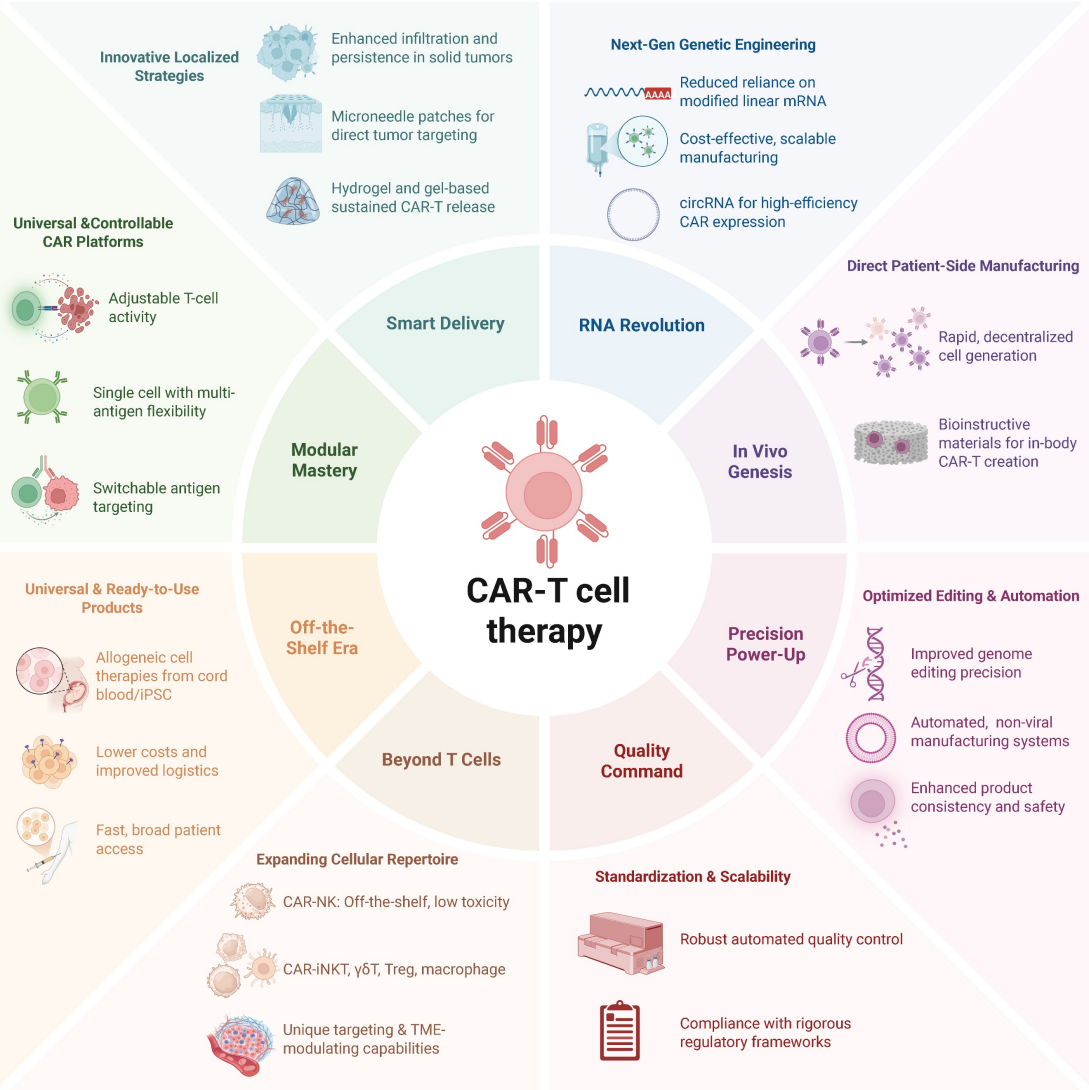
** Novel frontiers of CAR-T cell therapy.** The next-generation CAR-T combines modular, off-the-shelf cell platforms and expanded effector repertoires (e.g. CAR-NK, iNKT, macrophages) with smart localized delivery, RNA-based and in vivo manufacturing, precision genome editing and automation, and rigorous quality and regulatory controls—all aimed at making CAR-T therapy safer, more scalable, broadly accessible, and effective against solid tumors.

Ensuring the sustained activity of CAR-Ts *in vivo* is crucial for achieving long-term therapeutic outcomes. Innovative approaches, such as the development of universal immune receptor platforms like OmniCAR, allow for controllable T-cell activity and multi-antigen targeting with a single cell product (Figure 5). This modular system enables on-demand and adjustable T-cell responses, potentially enhancing persistence and efficacy, especially in solid tumors. ​While the modularity of OmniCAR allows for interchangeable targeting domains, the clinical feasibility and dosing kinetics of tag-based control is not yet well studied. In addition, overcoming the challenges posed by solid tumors requires novel delivery strategies. Localized delivery mechanisms, such as gel-based systems and microneedle patches, have been developed to enhance CAR-T cell infiltration and activity within solid tumor environments. For example, hydrogel-based delivery systems have been engineered to sustain CAR-T cell release at tumor sites, improving therapeutic efficacy. Innovations in RNA-based delivery systems, notably circRNA, have improved the efficiency, precision, and cost-effectiveness of manufacturing edited CAR-Ts. The ability of circRNA to mediate enhanced translation efficiency without expensive modified linear mRNA production presents a scalable and cost-effective alternative suitable for clinical-scale production 176.

Innovations in *in vivo* CAR-T cell manufacturing strategies are being explored to streamline production and reduce costs. Bioinstructive materials and rapid generation techniques aim to produce CAR-Ts directly within the patients, potentially overcoming current logistical challenges. Looking forward, the field must address critical challenges such as further optimization of editing precision, improved scalability, standardization of automated manufacturing processes, and rigorous regulatory frameworks to ensure product consistency and safety. Integrating automated, non-viral closed-system manufacturing and comprehensive quality control procedures will likely enable broader and safer clinical applications of CAR-Tct, ultimately facilitating their accessibility and affordability as UCAR-T therapeutics.

Expanding the application of CAR to include cell types beyond T cells introduces a novel dimension of therapeutic versatility. CAR-NK cells, derivable from cord blood or induced pluripotent stem cells (iPSCs), emerge as promising candidates for allogeneic, off-the-shelf products 177. In contrast with T-cell counterparts, CD19-targeted CAR-NK cells have shown potent tumor-lytic effects in patients with B-cell lymphoid tumors, without inducing CRS, neurotoxicity, or GVHD 178. CRISPR-enabled reprogramming is yielding CAR-NK products with materially improved persistence and function in solid tumors. Genome-scale and targeted loss-of-function studies converge on cell-intrinsic inhibitory nodes whose disruption restores effector potency. For example, CREM has emerged as a transcriptional checkpoint induced by CAR and IL-15 signaling in NK cells. Knockout of CREM increases cytokine production and cytotoxicity and improves *in vivo* tumor control in preclinical models 179. In complementary, unbiased screens, MED12, CCNC, and ARIH2 were identified as actionable regulators whose deletion enhances cytotoxicity, cytokine production, and metabolic fitness of primary human NK cells and CAR-NK constructs across hostile TME conditions 180. Beyond reversing dysfunction, metabolic reprogramming is critical for durability in nutrient-poor and hypoxic niches. Engineering CAR-NK cells to secrete the de novo IL-2/IL-15 mimetic Neo-2/15 activates IL-2Rβγ to STAT5 and Akt and engages c-Myc and NRF1 programs, which raises mitochondrial output and sustains antitumor activity in solid-tumor models 181. Together, these strategies show how precise genetic editing can overcome intrinsic limitations of NK cells and advance potent, off-the-shelf therapies for solid cancers.

Moreover, pioneering CAR-Tct using unconventional T cells, such as γδT cells, iNKT cells, Treg cells, and even macrophages, have entered preliminary trials with encouraging results 182-184. For example, distinct from traditional T cells perceiving peptide antigens dependent on MHC molecules, iNKT cells recognize lipid-based antigens presented by the β2M-associated MHC I-like molecule CD1d, which signifies that the function of NKT cells is restricted by CD-1d rather than classical TCR-MHC interaction, thus reducing risk for GVHD 185, 186. In addition, iNKT cells traffic across tissues and impede the immune-repressive activity of TAMs and MDSCs, which are compromised to create tumor-friendly environment in the TME ecosystem. Notably, early clinical trials of CAR-NKT cells targeting GD2 have shown promising anti-tumor activity without GVHD 187, 188.

## 7. Perspectives

The next mainstream wave of gene‑edited CAR‑T will be defined by three converging trajectories. First, clinical development is pivoting from bespoke, ex‑vivo manufacturing to in‑vivo “cell programming,” which has achieved durable B‑cell depletion in non‑human primates without lymphodepletion and is now in first‑in‑human evaluation using targeted lipid nanoparticles to transiently install CARs in circulating T cells—an approach that promises repeat‑dosing, dose titration, and step‑change scalability 189, 190. Second, universal allogeneic backbones are maturing from concept to practice: multiplex hypoimmune editing (e.g., TRAC/B2M/CIITA disruption with CD47 or HLA‑E stealth) is beginning to demonstrate evasion of host immunity in patients, supporting UCAR-T products with improved persistence in immunocompetent hosts 191. In solid tumors, specificity will increasingly depend on logic‑gated recognition, exemplified by synNotch‑primed GBM cells (E‑SYNC) now in the clinic and Tmod™ circuits (e.g., A2B530) that require tumor antigen and HLA loss‑of‑heterozygosity 192; early-phase readouts, together with intraventricular CARv3‑TEAM‑E producing dramatic—but initially transient—regressions in recurrent GBM, argue that multi‑antigen logic plus regional delivery will become the default template for high‑risk sites 193. Third, indication expansion into autoimmunity is accelerating and likely to normalize short‑course, drug‑free remissions: early CD19‑CAR‑T programs (e.g., CABA‑201) show encouraging activity, while allogeneic hypoimmune CAR‑T is advancing in autoimmune cohorts and has already induced remission in refractory SLE; iPSC‑derived products are entering the same space and may decouple supply from donor variability. These pushes will proceed under tighter safety governance—the FDA's classwide boxed warning on secondary T‑cell malignancies underscores the need for lifelong surveillance and will accelerate adoption of low‑break editors (base/prime) and layered control switches in next‑gen designs. Finally, industrialization (end‑to‑end automation, closed systems, and distributed “smart factories”) is moving from pilots to multi‑year capacity agreements and real‑world manufacturing, positioning the field to reduce cost, cycle time, and batch variability at scale.

## Figures and Tables

**Table 1:** Comparisons between CRISPR/Cas9, base editing, and prime editing in terms of editing precision, efficiency, off-target effects, delivery mechanisms, and translational maturity.

Category	Cas9	Base editing	Prime editing
type of edits supported (knock-out, point mutation, small insertion)	knock-out via NHEJ or insert sequences; supports large gene knock-in (requires donor DNA template).	C→T (CBEs) or A→G (ABEs) base substitutions to introduce point mutations or stop codons (enabling functional gene knockout).	versatile small edits without DSBs: install all 12 possible point mutations and small insertions or deletion.
types of edits not supported	precise point mutations are not directly introduced without a repair template; unpredictable indel patterns from NHEJ.	cannot insert large sequences (no new DNA added).	Inefficient for large DNA insertions (>50 bp); edit size limited by pegRNA design.
sequence constraints (edit window)	PAM sequence near the target; Cas9 cuts ~3 bp from PAM.	Cas9-derived nickase, requires PAM near the target; editing window typically 4-8 nucleotides from PAM, depending on base editor.	PAM required; edit can occur up to ~30 bp from PAM site, determined by pegRNA extension.
knock-out efficiency (disrupting genes)	high efficiency for gene disruption: >70-80% knock-out in primary T cells; multiplex knock-outs are feasible with concurrent sgRNAs; triple knock-outs in T cells have been reported.	high efficiency, even for multiplexed knock-outs: simultaneous disruption of 3-4 genes in T cells at high rates (>80%). Base editing of a single gene can be very efficient.	lower efficiency for gene knockout or editing in primary T cells; typical editing rates for point mutations <10-30%, insertions/deletions generally <20%.
precision editing efficiency (installing specific point mutations or small sequences)	moderate to low*.* Precise insertion or base substitution via HDR is much less efficient than knockout via NHEJ in T cells.	high for eligible targets; 30-80% editing efficiency for single base substitutions at accessible sites.	variable and generally lower than base editing; efficiency highly dependent on target and pegRNA design.
multiplex editing capacity (multiple simultaneous edits)	capable of multiplexing. Triple knock-outs using Cas9 have been done in CAR-T cells in clinical and preclinical studies.	well-suited for multiplex editing. Quadruple base edits in T cells have been achieved with high efficiency.	not yet demonstrated for multiplex in primary T cells.
DNA-level off-target activity (unintended genomic changes)	gene disruptions or translocations or introduce unintended mutations or large deletions with partial homology to the gRNA.	off-target point mutations-both Cas9-dependent and Cas9-independent, mostly single-nucleotide substitutions.	low off-target DNA editing: do not induce substantial off-target mutations and minimal collateral damage.
RNA off-target activity (off-target editing of RNA)	none observed for Cas9.	modern base editors greatly reduced RNA editing activity.	no direct RNA editing activity.
chromosomal rearrangement risk (translocations, large deletions)	elevated when multiplexing.	minimal risk no detectable translocations in T cells with triple or quadruple base edits.	low risk, similar to base editing.
delivery formats (RNP, mRNA, viral vectors)	RNP is widely used ex vivo; alternatively, mRNA Cas9 can be electroporated with sgRNA; for in vivo or ex vivo uses, viral vectors like AAV or lentiviral vectors; Cas9 RNP electroporation for the clinical CAR-T manufacturing.	mRNA electroporation is the most common; in research, some have used viral delivery; RNP delivery is challenging but possible; non-viral, transient approaches (mRNA or RNP) are preferred.	more complex delivery due to larger size. DNA, mRNA, or RNP, but each is less straightforward than for Cas9 or base editors.
delivery efficiency in T cells (uptake and expression success)	high editing rates but with notable cell toxicity.	efficient uptake and editing with gentler on the cells' health.	currently inefficient.
clinical trial usage to date	most advanced-already in trials.	emerging in the clinic.	preclinical stage.
manufacturing scalability (suitability for large-scale CAR-T production)	scalable with existing methods.	similarly scalable.	not yet scalable in practice.
suitability for UCAR-T	enabled by multiplex knock-outs.	highly suitable and perhaps optimal for allogeneic CAR-T.	not currently practical for allogeneic use.

ABEs: adenine base editors; CBEs: cytosine base editors; DSBs: double-strand breaks; NHEJ: non-homologous end joining; PAM: protospacer adjacent motif; pegRNA: prime editing guide RNA; RNP: ribonucleoprotein.

**Table 2:** Key clinical trials involving gene-edited CAR-T products

Trials products	Diseases	CAR Target	Editing tools	Key genes edited	Delivery Method	Clinical Phase	Ref
CTX110 (Allogeneic)	R/R LBCL	CD19	Cas9	TRAC, B2M (knockout)	Cas9 RNP electroporation	Phase I	165, 166
CTX120 (Allogeneic)	R/R MM	BCMA	Cas9	TRAC (CAR insertion), B2M (KO)	Cas9 RNP + rAAV6 electroporation	Phase I	8
CTX130 (Allogeneic)	R/R T-ALL, RCC	CD70	Cas9	TRAC (CAR insertion), B2M/CD70 (KO)	Cas9 RNP + rAAV6 electroporation	Phase I	167
CB-010 (Allogeneic)	R/R B-NHL	CD19	Cas9	TRAC (CAR insertion), PDCD1 (KO)	Cas9 RNP + rAAV6 electroporation	Phase I	168
CB-011 (Allogeneic)	R/R MM	BCMA	Cas12a	TRAC (CAR insertion), B2M (HLA-E knock-in)	Cas12a RNP + rAAV6 electroporation	Phase I	169
TT52CAR19 (Allogeneic)	Pediatric R/R B-ALL	CD19	Cas9	TRAC/CD52 (knockout)	Lentiviral vector (CAR+sgRNA)+Cas9 electroporation	Phase I	170
GC008t (MPTK-CAR-T) (Autologous)	mesothelin-positive solid tumors	Mesothelin	Cas9	PDCD1, TRAC (knockout)	Cas9 RNP	Phase I	171
PD1-19bbz (BRL-201) (Autologous)	R/R B-NHL	CD19	Cas9	PDCD1 (site-specific CAR knock-in)	Cas9 RNP + dsDNA	Phase I	172
BE-CAR7 (Allogeneic)	R/R T-ALL	CD7	CBE	TRBC/CD7/CD52 (inactivation by base edit)	Base editor mRNA	Phase I	119
CTX112 (Next-gen allogeneic)	R/R BCL	CD19	Cas9	TRAC/B2M/ *TGFBR2/ ZC3H12A*	AAV	Phase I/II	173
BEAM‑201	R/R T-ALL or T-LL	CD7	ABE	TRAC/CD7/CD52/PD1 (quadruple edits)	Base-editor mRNA	Phase I/II	174
TYU19 (SLE trial)	SLE (autoimmune)	CD19	Cas9	TRAC (allogeneic donor cells)	Cas9 RNP electroporation	Phase I	175

R/R B-ALL, relapsed/refractory B-cell acute lymphoblastic leukemia; RCC, renal cell carcinoma; R/R BCL, relapsed/refractory B-cell malignancies; R/R B-NHL, relapsed/refractory B-cell non-Hodgkin lymphoma; R/R LBCL, relapsed/refractory large B-cell lymphoma; R/R MM, relapsed/refractory multiple myeloma; R/R T-ALL, relapsed/refractory T-cell acute lymphoblastic leukemia; SLE, systemic lupus erythematosus; T-LL, T-cell lymphoblastic lymphoma.

## Abbreviations

ABEs: adenine base editors
ACT: adoptive cell transfer
AD: antigen domains
CAR: chimeric antigen receptor
CAR-Tct: CAR-T cell therapy
CAR-Ts: CAR-T cells
CBEs: cytosine base editors
CCRs: chemokine receptors
CD8Ts: CD8+ T cells
CDK4/6i: CDK4/6 inhibitors
circBE: circular BE RNA
CRISPRa: CRISPR activation
CRISPRi: CRISPR interference
CRS: cytokine release syndrome
CTLs: CD8+ cytotoxic T lymphocytes
dCas9: nuclease-deficient Cas9
DIC: disseminated intravascular coagulation
EGFR: epithelial growth factor receptor
GOF: gain-of-function
GRNs: gene regulatory networks
GVHD: graft-versus-host disease
HD: hinge domain
HSCs: hematopoietic stem cells
HSCT: hematopoietic stem cell transplantation
ICAHT: immune effector cell-associated hematotoxicity
ICANS: immune effector cell-associated neurotoxicity syndrome
iCasp9: inducible caspase 9
ICB: immune checkpoint blockade
ICIs: immune checkpoint inhibitors
iPSCs: induced pluripotent stem cells
ITAMs: immunoreceptor tyrosine-based activation motifs
LC: lung cancer
LOF: loss-of-function
MDSCs: myeloid-derived suppressor cells
MHC: major histocompatibility complex
MMP: matrix metalloproteinase
nCas9: Cas9 nickase
OTOT: on-target off-tumor
RIPKs: receptor-interacting protein kinases
scCRISPR: single-cell CRISPR
scRNA-seq: single-cell RNA sequencing
SD: signaling domain
SPMs: second primary malignancies
synNotch: synthetic Notch
TAAs: tumor-associated antigens
T-ALL: T-cell acute lymphoblastic leukemia
TAMs: tumor-associated macrophages
TANs: tumor-associated neutrophils
tanCAR-Ts: tandem CD19/CD20 CAR-T cells
Tex: exhausted T cells
TME: tumor microenvironment
TMD: transmembrane domain
TNF: tumor necrosis factor
Tpex: precursor exhausted T cells
Tregs: regulatory T cells
UCAR-T: universal CAR-T
